# Connective tissue growth factor is correlated with peritoneal lymphangiogenesis

**DOI:** 10.1038/s41598-019-48699-9

**Published:** 2019-08-21

**Authors:** Hiroshi Kinashi, Naohiro Toda, Ting Sun, Tri Q. Nguyen, Yasuhiro Suzuki, Takayuki Katsuno, Hideki Yokoi, Jan Aten, Masashi Mizuno, Shoichi Maruyama, Motoko Yanagita, Roel Goldschmeding, Yasuhiko Ito

**Affiliations:** 1 Department of Nephrology and Rheumatology, Aichi Medical University, Nagakute, Japan; 2 Department of Pathology, University Medical Center Utrecht, Utrecht, The Netherlands; 3 Department of Nephrology, Graduate School of Medicine, Kyoto University, Kyoto, Japan; 4 Department of Nephrology and Renal Replacement Therapy, Nagoya University Graduate School of Medicine, Nagoya, Japan; 5 Department of Pathology, Academic Medical Center, University of Amsterdam, Amsterdam, The Netherlands

**Keywords:** Kidney, Peritoneal dialysis

## Abstract

Lymphatic absorption in the peritoneal cavity may contribute to ultrafiltration failure in peritoneal dialysis (PD). Lymphatic vessels develop during PD-related peritoneal fibrosis. Connective tissue growth factor (CTGF, also called CCN2) is an important determinant of fibrotic tissue remodeling, but little is known about its possible involvement in lymphangiogenesis. In this study, we investigated the relationship between CTGF and peritoneal lymphangiogenesis. A positive correlation was observed between vascular endothelial growth factor-C (VEGF-C), a major lymphangiogenic growth factor, and the CTGF concentration in human PD effluents. CTGF expression was positively correlated with expression of lymphatic markers and VEGF-C in human peritoneal biopsies. We found a positive correlation between the increase in CTGF and the increase in VEGF-C in cultured human peritoneal mesothelial cells (HPMCs) treated with transforming growth factor-β1 (TGF-β1). The diaphragm is a central player in peritoneal lymphatic absorption. CTGF expression was also correlated with expression of VEGF-C and lymphatics in a rat diaphragmatic fibrosis model induced by chlorhexidine gluconate (CG). Furthermore, CTGF gene deletion reduced VEGF-C expression and peritoneal lymphangiogenesis in the mouse CG model. Inhibition of CTGF also reduced VEGF-C upregulation in HPMCs treated with TGF-β1. Our results suggest a close relationship between CTGF and PD-associated lymphangiogenesis.

## Introduction

Peritoneal dialysis (PD) is one type of renal replacement therapy for patients with end-stage renal disease (ESRD). Dialysate in the peritoneal cavity removes waste products by diffusion through peritoneal capillaries. Excess fluids are also removed by osmotic agents such as hypertonic glucose in the dialysate. Water removal (or ultrafiltration) equals transcapillary water removal minus lymphatic absorption^1^ because peritoneal lymphatic vessels continuously drain intraperitoneal fluids and transport them to the circulation^2^.

Ultrafiltration failure (UFF) accompanied by high peritoneal solute transport is an important complication seen after long-term PD. UFF is a main reason for the discontinuation of PD treatment, and is also associated with poor survival of PD patients^3–6^. The characteristic findings of chronic peritoneal damage in PD treatment are submesothelial fibrosis and neoangiogenesis^7,8^. However, the relationship between peritoneal fibrosis and UFF is still unclear. One potential mechanism focuses on lymphangiogenesis.

Clinical studies have shown that higher lymphatic absorption is associated with lower effective ultrafiltration^9^ and UFF^10^. However, the results from these clinical approaches are controversial^11,12^, and the process of lymphangiogenesis remains poorly understood. We therefore investigated whether lymphangiogenesis is associated with peritoneal fibrosis^13^. Signaling via vascular endothelial growth factor (VEGF)-C/D and VEGF receptor (VEGFR)-3 is central to lymphangiogenesis^14,15^. During peritoneal fibrosis, transforming growth factor–β (TGF-β) promotes VEGF-C expression, which leads to lymphangiogenesis^13,16^. Furthermore, blocking lymphangiogenesis with soluble VEGFR-3 improves impaired ultrafiltration in a mouse peritoneal fibrosis model^17^.

Connective tissue growth factor (CTGF) is a member of the CCN (CTGF/Cyr61/Nov) family and is an important player in the pathogenesis of fibrotic disorders. CTGF is increased in human PD effluents and human peritoneal biopsy samples in association with a high peritoneal solute transport rate^18^. CTGF production by human peritoneal mesothelial cells (HPMCs) is regulated by advanced glycation end products, glucose degradation products, and TGF-β^18–20^. In addition to the major regulatory role during fibrosis, CTGF is an important regulator of angiogenesis^21^. However, little is known about the possible association of CTGF with lymphangiogenesis.

We recently reported that CTGF promotes VEGF-C expression, which leads to fibrosis-related renal lymphangiogenesis^22^. The role of renal lymphangiogenesis seems to depend on the etiology of kidney diseases^23–25^. The role of peritoneal lymphatics in the physiology of PD is continuous absorption of intraperitoneal dialysate leading to a decrease in the ultrafiltration volume. Therefore, clarification of the mechanism of fibrosis-associated peritoneal lymphangiogenesis may lead to development of new therapeutic strategies for UFF.

In this study, we investigated the relationship between CTGF and VEGF-C expression in human PD effluents. We also explored the association of CTGF with VEGF-C and lymphatic vessels in human peritoneal biopsy samples. *In vitro*, we analyzed TGF-β1-induced CTGF and VEGF-C upregulation in HPMCs collected from spent PD effluents. In addition, we analyzed lymphangiogenesis in the diaphragm, which we previously showed to play a central role in peritoneal lymphatic absorption^13,17^. This was done in the chlorhexidine gluconate (CG)-induced rat peritoneal fibrosis model^13,26^. To explore the role of CTGF in peritoneal lymphangiogenesis, we assessed the effect of CTGF reduction on peritoneal lymphangiogenesis using CTGF conditional knockout (CTGF^−/−^) mice^27,28^. We also investigated the effect of CTGF inhibition on TGF-β1-induced VEGF-C upregulation in HPMCs using CTGF small interfering RNA (siRNA).

## Results

### Positive correlation between CTGF and VEGF-C expression in human PD effluents

**Figure 1:**
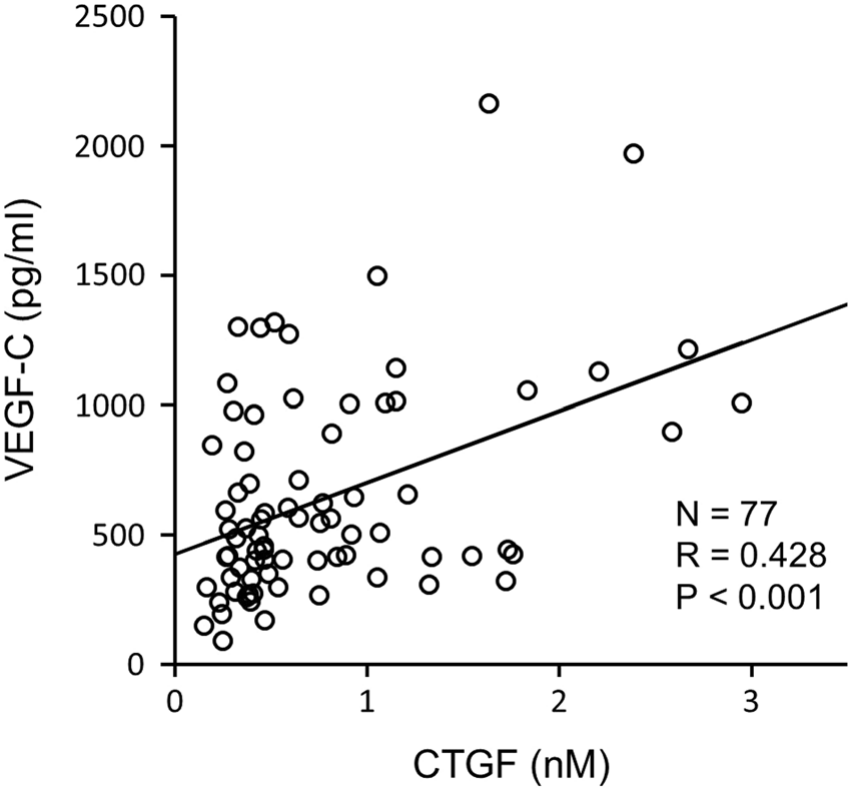
Positive correlation between connective tissue growth factor (CTGF) and vascular endothelial growth factor-C (VEGF-C) expression in human peritoneal dialysis (PD) effluents. CTGF and VEGF-C protein levels in 77 overnight dwelled human PD effluents were measured with a sandwich enzyme-linked immunosorbent assay. The CTGF concentration was positively correlated with the VEGF-C concentration in human PD effluents. Pearson correlation was used for the analysis.

To explore the involvement of CTGF in peritoneal lymphangiogenesis, we measured the CTGF and VEGF-C concentration in the overnight dwelled PD effluents derived from 77 patients. The mean CTGF concentration was 0.79 ± 0.63 (range = 0.16–2.95) nM. The mean VEGF-C concentration was 643 ± 407 (range = 89.3–2161) pg/ml. A positive correlation was observed between the CTGF and VEGF-C concentration in the PD effluents (R = 0.428, P < 0.001, Fig. 1).

### CTGF expression was correlated with expression of lymphatics and VEGF-C in human peritoneal biopsy samples

**Figure 2:**
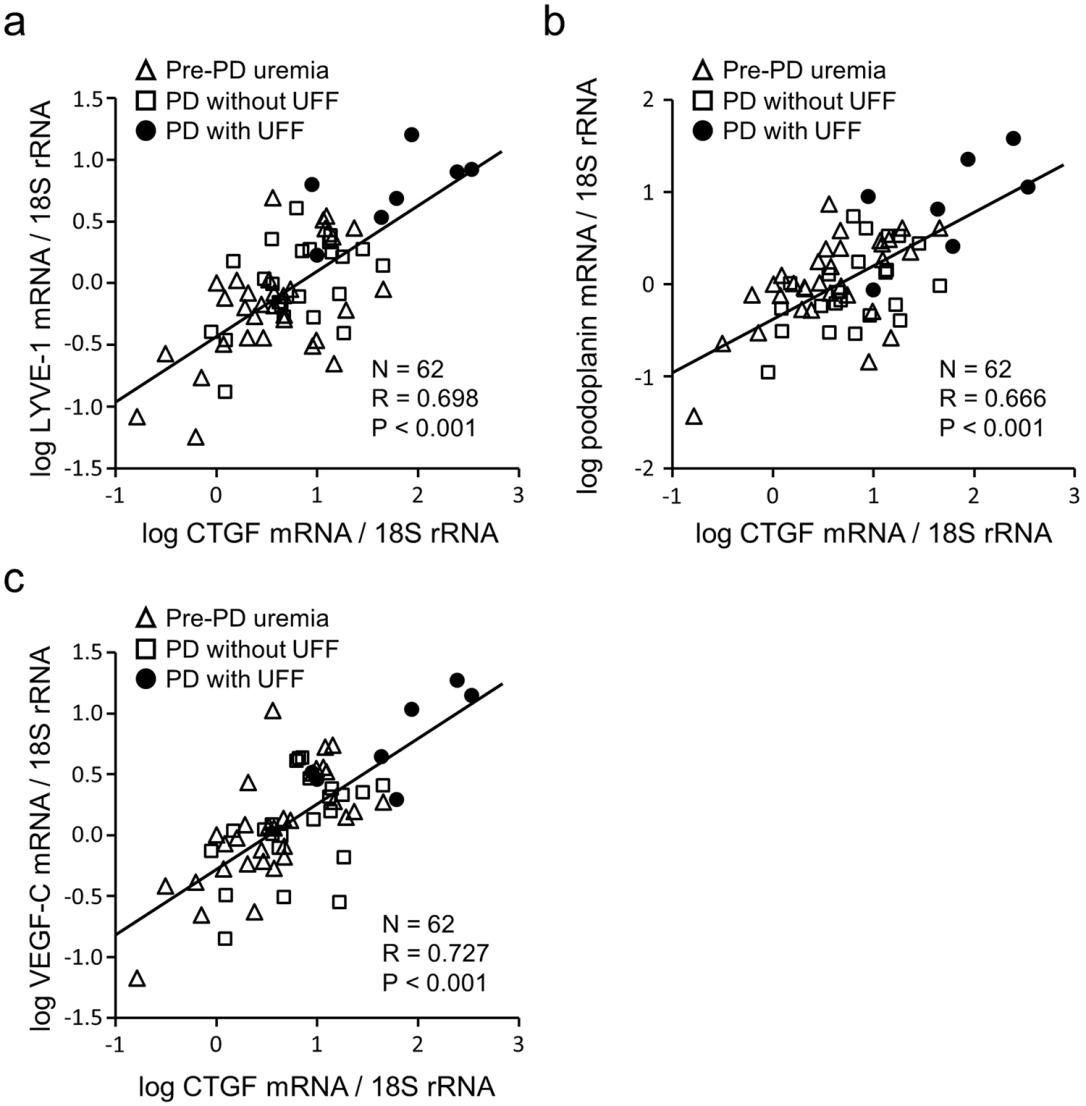
Connective tissue growth factor (CTGF) expression was correlated with expression of lymphatic markers and vascular endothelial growth factor-C (VEGF-C) in human peritoneal biopsies. Human peritoneal biopsy specimens were collected from 32 uremic patients before initiation of peritoneal dialysis (PD), 23 patients undergoing PD not complicated by ultrafiltration failure (UFF), and seven PD patients with UFF. Quantitative polymerase chain reaction analysis showed positive correlations between CTGF messenger RNA (mRNA) expression and lymphatic vessel endothelial hyaluronan receptor-1 (LYVE-1) (**a**), podoplanin (**b**), and VEGF-C (**c**) mRNA expression in 62 human peritoneal biopsy samples. 18 S ribosomal RNA (rRNA) was used as an internal reference. Values were transformed into the logarithmic scale for Pearson correlation.

Second, we analyzed the messenger RNA (mRNA) expression of CTGF, VEGF-C, and lymphatic markers (lymphatic vessel endothelial hyaluronan receptor-1 (LYVE-1) and podoplanin) in 62 human peritoneal biopsy samples derived from pre-dialysis uremic patients (n = 32) and PD patients with or without UFF (n = 7, n = 23, respectively). CTGF mRNA expression was positively correlated with LYVE-1 (R = 0.698, P < 0.001, Fig. 2a), podoplanin (R = 0.666, P < 0.001, Fig. 2b), and VEGF-C (R = 0.727, P < 0.001, Fig. 2c) mRNA expression in the human peritoneal membranes.

We also analyzed the correlations between the mRNAs in each category. CTGF mRNA expression was similarly correlated with LYVE-1 (R = 0.571, P < 0.01; R = 0.476, P < 0.05, Supplementary Figs 1a and 2a), podoplanin (R = 0.577, P < 0.01; R = 0.500, P < 0.05, Supplementary Figs 1b and 2b), and VEGF-C (R = 0.713, P < 0.001; R = 0.478, P < 0.05, Supplementary Figs 1c and 2c) mRNA expression in the peritoneum of pre-PD patients and in the peritoneum of PD patients without UFF, respectively. CTGF mRNA expression tended to be correlated with LYVE-1 and podoplanin mRNA expression in the peritoneum of PD patients with UFF, but the relationship was not statistically significant (Supplementary Fig. 3a,b). However, CTGF mRNA expression was significantly correlated with VEGF-C mRNA expression in the UFF peritoneum (R = 0.781, P < 0.05, Supplementary Fig. 3c).

In addition, we investigated representative human peritoneal biopsies derived from a pre-PD patient and a PD patient with UFF by performing immunohistochemistry (IHC) for CTGF, VEGF-C, and a lymphatic marker (D2–40). Serial sections of the UFF peritoneum showed more D2-40-positive lymphatic vessels accompanied by increased expression of CTGF and VEGF-C than the pre-PD peritoneum (Supplementary Fig. 4).

### The increase in CTGF mRNA expression was correlated with the increase in VEGF-C mRNA expression in HPMCs treated with TGF-β1

**Figure 3:**
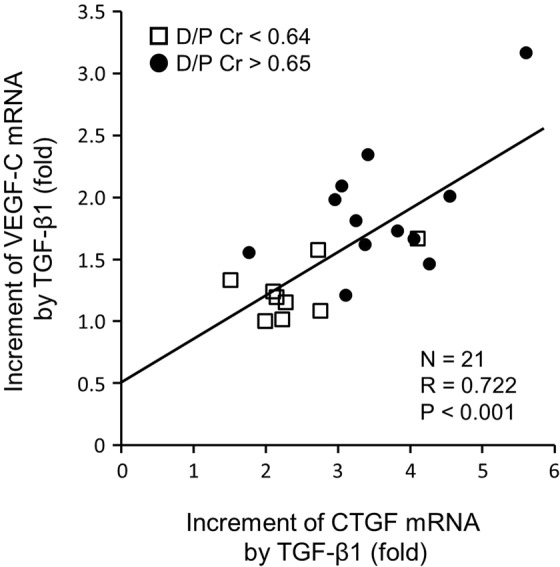
Positive correlation between the increase in connective tissue growth factor (CTGF) and the increase in vascular endothelial growth factor-C (VEGF-C) in human peritoneal mesothelial cells (HPMCs) treated with transforming growth factor-β1 (TGF-β1). HPMCs were collected from the spent peritoneal dialysis (PD) effluents derived from 21 PD patients with various peritoneal transport functions. Nine patients showed lower peritoneal permeability (dialysate to plasma ratio of creatinine, D/P Cr < 0.64), and the other 12 patients showed higher peritoneal permeability (D/P Cr > 0.65). Cultured HPMCs were treated with 5 ng/ml recombinant human TGF-β1 for 12 h and harvested. Increases in CTGF and VEGF-C messenger RNA (mRNA) following incubation with TGF-β1 were analyzed with quantitative polymerase chain reaction. A positive correlation was observed between CTGF and VEGF-C mRNA amplification in HPMCs treated with TGF-β1. 18 S ribosomal RNA (rRNA) was used as an internal reference. Pearson correlation was used for the analysis.

Next, we assessed CTGF and VEGF-C mRNA expression in HPMCs derived from 21 PD patients with different peritoneal membrane transport functions. Cells were cultured with or without TGF-β1 (5 ng/ml) and harvested 12 h later. CTGF and VEGF-C mRNA expression was increased to varying degrees by TGF-β1 treatment. A positive correlation was observed between the fold-increase in CTGF and the fold-increase in VEGF-C mRNA (R = 0.722, P < 0.001, Fig. 3) after TGF-β1 treatment.

### CTGF expression was correlated with expression of VEGF-C and lymphatic vessels in the rat CG-induced diaphragmatic fibrosis model

**Figure 4:**
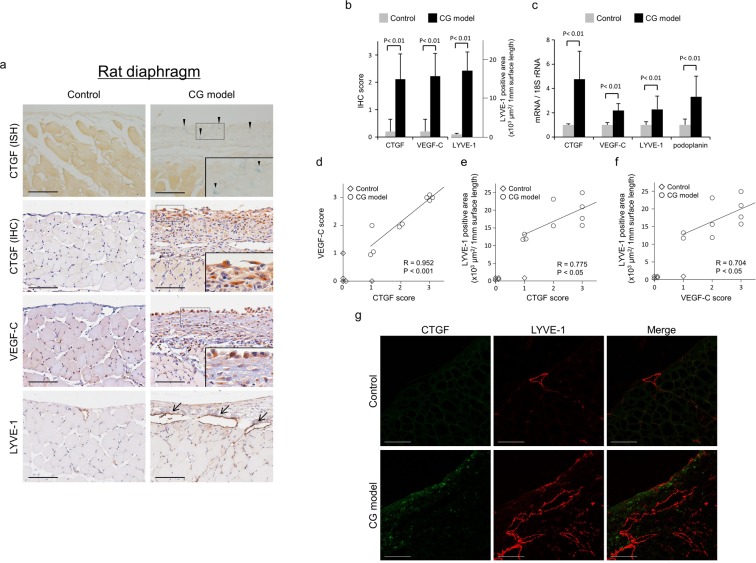
Connective tissue growth factor (CTGF) expression was correlated with expression of vascular endothelial growth factor-C (VEGF-C) and lymphatics in a rat diaphragmatic fibrosis model induced by chlorhexidine gluconate (CG). Diaphragmatic fibrosis was induced by intraperitoneal injection of CG in rats. Control rats were treated with saline. (**a**,**b**) Immunohistochemistry (IHC) showed that expression of CTGF, VEGF-C, and lymphatic vessel endothelial hyaluronan receptor-1 (LYVE-1) was increased in the rat diaphragmatic fibrosis model induced by CG compared with controls. The staining intensity for CTGF and VEGF-C was scored as follows: 0, absent; 1, mild; 2, moderate; 3, extensive. CTGF messenger RNA (mRNA) was detected in the CG model with *in situ* hybridization (ISH). Arrowheads indicate CTGF mRNA expression. Arrows indicate LYVE-1-positive lymphatic vessels. Insets show magnification of the dotted-line boxed areas. Scale bars; 100 μm. (**c**) CTGF, VEGF-C, LYVE-1, and podoplanin mRNA expression were increased in the diaphragm in CG-injected rats compared with controls as seen with quantitative polymerase chain reaction analysis. (**d**–**f**) IHC analysis showed positive correlations among CTGF, VEGF-C, and LYVE-1 expression in the CG model. Spearman’s rank correlation was used for the analysis. (**g**) Double immunofluorescent staining showed that CTGF expression was increased around LYVE-1-positive lymphatic vessels in the CG model. Bar graphs show means ± SD (Control, n = 5; CG model, n = 9).

IHC analysis showed that expression of CTGF (P < 0.01), VEGF-C (P < 0.01), and LYVE-1-positive lymphatic vessels (P < 0.01) was increased in the diaphragm in rats intraperitoneally injected with CG (n = 9) compared with control rats treated with saline (n = 5) (Fig. 4a,b, Supplementary Fig. 5). In addition, increased expression of CTGF mRNA was observed in the CG model with *in situ* hybridization (ISH) (Fig. 4a). Quantitative polymerase chain reaction (qPCR) analysis showed that mRNA expression of CTGF, VEGF-C, LYVE-1, and podoplanin was increased 4.8- (P < 0.01), 2.2- (P < 0.01), 2.3- (P < 0.01), and 3.3-fold (P < 0.01), respectively, in the diaphragm in CG-injected rats compared to controls (Fig. 4c). Quantification of IHC showed that CTGF expression was positively correlated with expression of VEGF-C (R = 0.952, P < 0.001, Fig. 4d), and LYVE-1-positive lymphatic vessels (R = 0.775, P < 0.05, Fig. 4e) in the rats injected with CG. Moreover, a positive correlation was observed between VEGF-C and LYVE-1 expression in these rats (R = 0.704, P < 0.05, Fig. 4f). Additionally, double immunofluorescent staining of the CG-treated diaphragm showed more LYVE-1-positive lymphatic vessels in regions with increased expression of CTGF than in the control diaphragm (Fig. 4g).

### Genetic deletion of CTGF reduced lymphangiogenesis and VEGF-C expression in the mouse CG-induced peritoneal fibrosis model

**Figure 5:**
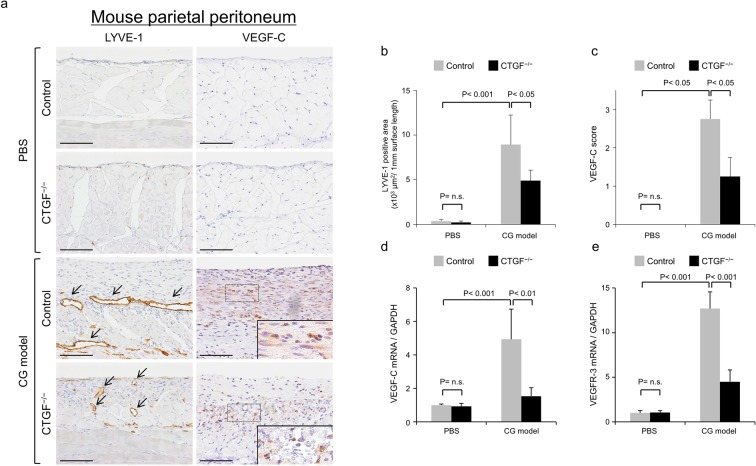
Genetic deletion of connective tissue growth factor (CTGF) reduced lymphangiogenesis and vascular endothelial growth factor-C (VEGF-C) expression in a mouse peritoneal fibrosis model induced by chlorhexidine gluconate (CG). Peritoneal fibrosis was induced by intraperitoneal injection of CG in wild-type mice (Control) and CTGF knockout (CTGF^−/−^) mice. PBS-treated mice were used for comparison. (**a**–**c**) Immunohistochemical analysis showed that the increased expression of lymphatic vessel endothelial hyaluronan receptor-1 (LYVE-1) and VEGF-C in the CG model was significantly decreased in CTGF^−/−^ mice compared with control mice. We found no significant differences in LYVE-1 and VEGF-C expression between PBS-treated control peritoneum and PBS-treated CTGF^−/−^ peritoneum. The staining intensity for VEGF-C was scored as follows: 0, absent; 1, mild; 2, moderate; 3, extensive. Arrows indicate LYVE-1-positive lymphatic vessels. Insets show magnification of dotted-line boxed areas. Scale bars; 100 μm. (**d**,**e**) Quantitative polymerase chain reaction analysis showed that the increased expression of VEGF-C and VEGF receptor-3 (VEGFR-3) messenger RNA (mRNA) in the CG model was significantly decreased in CTGF^−/−^ mice compared with control mice. We found no significant differences in VEGF-C or VEGFR-3 mRNA expression between the PBS-treated control peritoneum and PBS-treated CTGF^−/−^ peritoneum. Glyceraldehyde-3-phosphate dehydrogenase (GAPDH) was used as an internal reference. Graphs show means ± SD (n = 4 for each group). n.s.; not significant.

We investigated the effect of genetic deletion of CTGF on CG-induced peritoneal lymphangiogenesis using tamoxifen-inducible conditional CTGF^−/−^ mice. IHC of the parietal peritoneum showed that LYVE-1-positive lymphatic vessels and VEGF-C expression were barely present in the phosphate-buffered saline (PBS)-treated peritoneum (Fig. 5a). In contrast, dilated lymphatic vessels appeared and VEGF-C expression was increased in CG-induced peritoneal fibrosis (Fig. 5a). IHC analysis showed that the LYVE-1-positive area and VEGF-C score (Supplementary Fig. 6) were significantly increased in the CG-treated control peritoneum compared with the PBS-treated control peritoneum (P < 0.001, P < 0.05, respectively, Fig. 5b,c), and were significantly decreased in the CG-treated CTGF^−/−^ peritoneum compared with the CG-treated control peritoneum (P < 0.05, Fig. 5b,c). We found no significant differences in the LYVE-1-positive area and VEGF-C score between the PBS-treated control peritoneum and PBS-treated CTGF^−/−^ peritoneum (Fig. 5b,c). qPCR analysis showed that VEGF-C and VEGFR-3 mRNA were increased 4.9- (P < 0.001) and 12.7-fold (P < 0.001), respectively, in the CG-treated control peritoneum compared with the PBS-treated control peritoneum. The CG-treated CTGF^−/−^ peritoneum showed a significant decrease in both VEGF-C and VEGFR-3 mRNA compared with the CG-treated control peritoneum (P < 0.01, P < 0.001, respectively, Fig. 5d,e). We found no significant differences in VEGF-C or VEGFR-3 mRNA expression between the PBS-treated control peritoneum and the PBS-treated CTGF^−/−^ peritoneum (Fig. 5d,e). Double immunofluorescent staining showed that the expression pattern of VEGFR-3-positive lymphatic vessels was similar to the expression pattern of LYVE-1-positive lymphatic vessels in the CG-treated control peritoneum and CG-treated CTGF^−/−^ peritoneum (Supplementary Fig. 7).

### siRNA-mediated knockdown of CTGF reduced VEGF-C expression in HPMCs treated with TGF-β1

**Figure 6:**
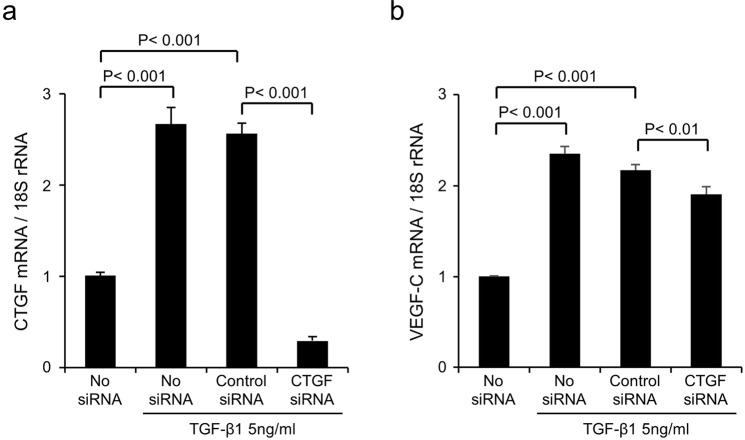
Small interfering RNA (siRNA)-mediated knockdown of connective tissue growth factor (CTGF) reduced vascular endothelial growth factor-C (VEGF-C) upregulation in human peritoneal mesothelial cells (HPMCs) treated with transforming growth factor-β1 (TGF-β1). HPMCs were collected from the peritoneal dialysis (PD) effluent derived from a PD patient with high peritoneal permeability. CTGF messenger RNA (mRNA) expression was inhibited by siRNA in cultured HPMCs treated with 5 ng/ml recombinant human TGF-β1 for 24 h. Quantitative polymerase chain reaction analysis showed that the TGF-β1-induced mRNA upregulation of both CTGF and VEGF-C was reduced by CTGF siRNA compared with control siRNA. 18 S ribosomal RNA (rRNA) was used as an internal reference. Graphs show means ± SD (n = 3 for each group).

Finally, we assessed the effect of siRNA-mediated knockdown of CTGF on VEGF-C expression in HPMCs. We inhibited CTGF expression with CTGF siRNA in HPMCs treated with TGF-β1. CTGF and VEGF-C mRNA expression was increased 2.7- (P < 0.001) and 2.4-fold (P < 0.001), respectively, in HPMCs after 24 h of incubation with TGF-β1 (Fig. 6a,b). CTGF siRNA significantly reduced TGF-β1-stimulated CTGF and VEGF-C expression (P < 0.001, P < 0.01, respectively) compared with negative control siRNA (Fig. 6a,b).

## Discussion

Involvement of lymphatic absorption in the physiology of PD was noticed three decades ago^1^. Clinical studies showed that higher lymphatic absorption is associated with PD duration and UFF^9,29^. However, the effective lymphatic absorption rate, which was estimated by the disappearance of intraperitoneally administered macromolecules, such as radioactive iodinated serum albumin or dextran, is controversial^11,12^. Lymphatics in PD have been less well studied in the recent decade. In contrast, owing to the recent discovery of several useful lymphatic markers and key molecular mechanisms, lymphangiogenesis has been studied in a variety of diseases, such as tumor metastasis^30^, inflammatory disease^31^, heart disease^32^, and transplant rejection^33^. We observed lymphangiogenesis in various types of human kidney diseases, and demonstrated that the number of lymphatic vessels is correlated with the degree of renal interstitial fibrosis^34^. Lymphangiogenesis is also associated with human peritoneal fibrosis, and patients with UFF show high expression of lymphatic vessel markers in the peritoneum compared to PD patients without UFF^13^. Interestingly, investigations using the rat remnant kidney model recently revealed that chronic kidney disease itself can induce peritoneal fibrosis, lymphangiogenesis, and a high lymphatic absorption rate, independent of exposure to PD solution^35^. Two major mechanisms, i.e., neoangiogenesis accompanied by high vascular permeability, and reduction of the osmotic gradient by tissue fibrosis, are highlighted in the context of UFF in peritoneal fibrosis^36^. However, fibrosis-associated lymphangiogenesis additionally suggests the possible involvement of lymphatic absorption in the UFF mechanism of progressive peritoneal membrane injury.

In this study, we first explored whether the CTGF concentration was positively correlated with the VEGF-C concentration in human PD effluents. Both CTGF and VEGF-C protein in human dialysate are correlated with peritoneal permeability^13,18,37^, suggesting that both could be biomarkers and therapeutic targets for UFF. The dialysate protein level is affected by local peritoneal production and transfer from the blood circulation, which is also dependent on several factors including the plasma level^38^, peritoneal protein clearance^39^, molecular weight with degradation^18^, and protein charge^40^. Although the data include complicated factors, our findings are compatible with a role for CTGF in lymphangiogenesis in the peritoneum.

CTGF was detected and present at higher levels in mesothelial cells and fibroblasts in human fibrotic peritoneal membranes in relation to UFF^18^. mRNA expression of CTGF, VEGF-C, and lymphatic markers in peritoneum is higher in UFF patients than in pre-dialysis uremia patients, and is correlated with peritoneal thickness^13,18^. Interestingly, no correlation is present between CTGF mRNA expression and the density of blood vessels in human peritoneal biopsies^18^. Unlike the blood vessel analysis, however, our current study showed that CTGF mRNA expression was positively correlated with VEGF-C, LYVE-1, and podoplanin mRNA expression in human peritoneal biopsies. Our results indicate that local CTGF expression in the context of peritoneal fibrosis is closely linked to lymphangiogenesis.

Mesothelial cells that line the surface of the peritoneal cavity predominantly regulate intraperitoneal homeostasis including the synthesis of cytokines, growth factors, and matrix proteins^41^. In parallel with the enhancement of profibrotic activity by CTGF production, mesothelial cells also play an important role in lymphangiogenesis by producing VEGF-C. We treated HPMCs from spent PD effluent with TGF-β1, which can be elevated in the peritoneal cavity by several mechanisms, such as exposure to dialysate glucose^42^, advanced glycation end products^43^, glucose degradation products^20^, and the presence of bacterial peritonitis^44^. Our results demonstrated that production of both CTGF and VEGF-C was increased by TGF-β1 to varying degrees in HPMCs from individual patients, and that their enhancement showed a positive correlation. This is in accordance with the findings in PD effluent and peritoneal biopsy analyses.

The diaphragm contains a specialized form of the lymphatic absorption system including lymphatic lacunae and mesothelial stomata^2^. This aspect of UFF and lymphangiogenesis can be studied only in animal experiments. IHC of diaphragm sections from the rat CG model showed that CTGF is mainly increased in peritoneal mesothelial cells and fibroblast-like cells, and that VEGF-C is increased in mesothelial cells and mononuclear infiltrates, consistent with observations in the parietal peritoneum^13,18^. CTGF expression in the diaphragm is significantly correlated with expression of VEGF-C and LYVE-1-positive lymphatic vessels, supporting the concept of the involvement of CTGF in diaphragmatic lymphangiogenesis.

Several animal experiments demonstrated that CTGF inhibition ameliorates the development of fibrosis in obstructive nephropathy^45^, diabetic nephropathy^46^, allograft nephropathy^47^, and the remnant kidney model^48^. In addition, recent studies showed that CTGF inhibition ameliorates CG-induced peritoneal fibrosis through suppression of fibroblast accumulation, angiogenesis, and inflammation^28,49^. Furthermore, we recently reported that CTGF is involved in fibrosis-associated renal lymphangiogenesis through regulation of, and direct interaction with, VEGF-C^22^. CTGF knockout results in suppression of lymphangiogenesis in obstructive nephropathy and an ischemia reperfusion injury model, which is congruent with the effect of CTGF inhibition on peritoneal lymphangiogenesis in this study.

CTGF directly binds to VEGF-C and inhibits VEGF-C-induced growth of lymphatic endothelial cells^22^. Therefore, we suggest that the reduction in lymphangiogenesis in the CG-treated CTGF^−/−^ peritoneum was mediated by not the absence of CTGF but by decreased VEGF-C expression. However, the effect of CTGF inhibition on VEGF-C reduction was smaller *in vitro* than *in vivo*. Regarding this point, we noticed that the CG-treated CTGF^−/−^ peritoneum showed less TGF-β1 mRNA expression than the CG-treated control peritoneum^28^. TGF-β1 promotes VEGF-C expression in peritoneal mesothelial cells and macrophages as shown in our previous reports^13,50^. We suggest that the reduction in lymphangiogenesis in the CG-treated CTGF^−/−^ peritoneum is due to decreased TGF-β levels in addition to less VEGF-C induction by TGF-β due to little CTGF expression.

The specific blocking of VEGFR-3 signals using soluble VEGFR-3, a decoy receptor for VEGF-C and VEGF-D, significantly reduces lymphangiogenesis without altering inflammation or fibrosis in a mouse peritoneal fibrosis model induced by methylglyoxal, a toxic glucose degradation product^17^. In contrast, CTGF inhibition significantly reduces peritoneal fibrosis and inflammation in the mouse CG model^28^. Therefore, we propose that blocking VEGFR-3 signals specifically suppresses lymphangiogenesis in peritoneal fibrosis, which is different from the effect of CTGF inhibition.

In conclusion, we have identified a close association between CTGF expression and lymphangiogenesis in peritoneal fibrosis. Studies are underway to clarify the possible benefit of targeting CTGF to prevent lymphangiogenesis, UFF, and peritoneal fibrosis in the course of PD therapy.

## Methods

### Patient profiles

The studies with human samples were performed in accordance with the ethical guidelines of the 1975 Declaration of Helsinki and were approved by the Ethics Committee for Human Research of the Faculty of Medicine, Nagoya University (approval no. 298: peritoneal fluid experiment; approval no. 299: peritoneal tissue experiments). Informed consent was obtained from all patients.

CTGF and VEGF-C protein levels were measured in 77 overnight dwelled (8.97 ± 1.62 h) human peritoneal effluent samples (24 women and 53 men), which were collected at Nagoya University Hospital (Nagoya, Japan) and affiliated hospitals between July 2005 and April 2008^13,18^. The mean age of all patients was 54.7 ± 13.0 (range = 28–84) years, and the mean period of PD treatment was 32.1 ± 32.6 (range = 1–132) months. Diabetic nephropathy was the cause of ESRD in 26 PD patients (33.8%). No patients had the complication of peritonitis for over 1 month before the study, and patients with other diseases, such as liver or lung diseases and malignancy, were not included. Patients treated with combination therapy (hemodialysis + PD) were excluded from this study. Peritoneal transport was calculated by the dialysate-to-plasma ratio of creatinine (D/P Cr), and the average value was 0.68 ± 0.13 (range = 0.28–0.96).

A total of 62 peritoneal tissue samples were collected from 32 pre-dialysis chronic kidney disease patients at the time of PD catheter insertion and 30 PD patients at the time of PD catheter removal for PD-related complications. The mean age of pre-dialysis uremia patients (nine women and 23 men) was 61.3 ± 12.4 years. Among 30 PD patients, seven patients (four women and three men) were regarded as having UFF and needed to be treated with more than four hypertonic bags (2.27% glucose and 3.86% glucose or icodextrin) per day to maintain their fluid status^51^. The mean age of UFF patients was 55.9 ± 11.6 years, and the mean period of PD treatment was 9.4 ± 6.6 years. The other 23 PD patients (10 women and 13 men) had their catheters removed for reasons other than UFF, such as transplantation, mental disorders, severe exit site infection, or difficulty with performing bag exchanges. The mean age of the 23 patients without UFF was 60.8 ± 13.2 years, and the mean period of PD treatment was 3.7 ± 3.0 years. Correlations between CTGF and LYVE-1, podoplanin, and VEGF-C mRNA expression were evaluated^13,18^.

### Cell culture study

HPMCs were collected by centrifugation of PD effluents derived from 21 clinically stable patients using modified methods as described previously^13,18^. Nine patients (four women and five men) were classified into the low or low average category of the peritoneal equilibration test (D/P Cr < 0.64), and the other 12 patients (six women and six men) were classified into the high or high average category (D/P Cr > 0.65). Subconfluent cells plated in 6-cm dishes were rinsed with PBS, and the cultures were replaced with serum-free medium for 24 h for a quiescent state. Thereafter the cultures were incubated with or without 5 ng/ml recombinant human TGF-β1 (240-B, R&D Systems, Minneapolis, MN), which was mixed in serum-free medium. Cultures were harvested at 12 h (n = 3). All experiments were performed during the third to fourth passage. To explore the correlation between CTGF and VEGF-C upregulation by TGF-β1, we assessed the fold-increase in CTGF and VEGF-C mRNA treated with TGF-β1 compared with the basal mRNA expression without TGF-β1 treatment.

For the CTGF inhibition study, HPMCs derived from a patient with high peritoneal permeability were used. Cells were transfected with 10 nM silencer select siRNA for CTGF or a negative control (Ambion, Austin, TX) using Lipofectamine RNAiMAX (Invitrogen, Carlsbad, CA). After 24 h of transfection, culture medium was replaced with serum-free medium alone or medium with 5 ng/ml TGF-β1. Cells were harvested after 24 h of incubation (n = 3).

### Animal models

The rat study was performed in accordance with the Animal Experimentation Guidelines of Nagoya University Graduate School of Medicine (Nagoya, Japan), and was approved by the Animal Experimentation Committee of Nagoya University (approval no. 23325). Eight-week-old male Sprague-Dawley rats (Japan SLC, Hamamatsu, Japan) were used for the study. Nine rats were intraperitoneally injected every other day with 3 ml/200 g body weight of 0.04% CG (Wako, Japan) and 10% ethanol (Wako) dissolved in saline^13^. Five control rats were treated with the same dose of saline. All diaphragm samples were collected on day 16.

The mouse experiment was performed according to the guidelines of the Animal Experimentation Committee of Kyoto University (Kyoto, Japan), and was approved by the Animal Experimentation Committee of Kyoto University (approval no. MedKyo17174, MedKyo16526, MedKyo15250). ROSA26-ERT2CRE/floxCTGF mice were treated with tamoxifen to establish CTGF^−/−^ mice as previously described^27,28^. Mice were intraperitoneally injected three times a week with 0.3 ml of 0.1% CG in 15% ethanol dissolved in PBS^28,52^. The other mice were injected with the same dose of PBS for comparison. CG injection induces peritoneal fibrosis, inflammation, and neoangiogenesis^28^. All mice were euthanized after 4 weeks, and parietal peritoneal samples were collected (n = 4).

The harvested animal samples were analyzed with IHC, ISH, and qPCR.

### Enzyme-linked immunosorbent assay (ELISA)

CTGF protein was measured in human peritoneal dialysate samples with a sandwich ELISA using a previously described method with modifications^18^. Microtiter plates were coated overnight at 4 °C with a mouse monoclonal antibody (FibroGen, San Francisco, CA) that binds distinct epitopes in domain 2 of CTGF. Wells were rinsed and blocked with 1% bovine serum albumin overnight at 4 °C. After washing, the NH2-terminal fragment of human recombinant CTGF (FibroGen), which was used for the calibration curve, and samples were added and incubated with alkaline phosphatase-conjugated antibody against CTGF (Leinco Technologies, Fenton, MO) overnight at 4 °C. Plates were washed, and substrate solution containing p-nitrophenyl phosphate was added. Absorbance was read at 405 nm.

The VEGF-C concentration in human PD effluents was measured with the Human VEGF-C Assay Kit (IBL, Takasaki, Japan) according to the manufacturer’s instructions.

All samples for ELISA were evaluated in duplicate.

### qPCR

Separate samples were collected for RNA extraction. Total RNA was extracted from tissue and cultured cells using RNeasy columns (Qiagen, Hilden, Germany). After cDNA synthesis, samples were mixed with TaqMan Gene Expression Assays (CTGF, Hs00170014_m1, Rn01537279_g1; VEGF-C, Hs00153458_m1, Rn00586458_m1, Mm00437310_m1; LYVE-1, Hs00272659_m1, Rn01510422_m1; podoplanin, Hs00366764_m1, Rn00571195_m1; VEGFR-3, Mm01292604_m1; Applied Biosystems, Foster City, CA) that were run on Applied Biosystems Prism 7500HT. 18 S ribosomal RNA (4319413E) or TaqMan® Rodent GAPDH Control Reagents were used as an internal reference.

### IHC and ISH

IHC for CTGF (sc-14939; Santa Cruz Biotechnology Inc., Dallas, TX), VEGF-C (Zymed Laboratories, South San Francisco, CA), D2-40 (BioLegend, San Diego, CA), LYVE-1 (Acris Antibodies GmbH, Herfold, Germany), and VEGFR-3 (R&D Systems) was performed as previously reported^13,34,53^. To determine positive areas on stained sections, 10 random fields per section were chosen and photographed. LYVE-1-positive lymphatic vessels were identified and quantitated using Image J software, and the density was calculated for the analysis of lymphangiogenesis. CTGF and VEGF-C expression were semiquantitatively classified as follows: 0, absent; 1, mild; 2, moderate; 3, extensive^13,54^.

ISH to detect CTGF mRNA was performed on formalin-fixed paraffin-embedded rat diaphragm samples according to previously described methods^18,55^.

### Statistical analysis

We expressed statistical values as the mean ± standard deviation (SD). The Mann-Whitney U-test was used for the comparison between two independent groups. Variables of mRNA expression in human peritoneal biopsies were transformed into the logarithmic scale, and then a parametric test was performed. Ordinal CTGF and VEGF-C data in the CG model were compared using Spearman rank correlation. Pearson correlation was used for the other variables. All analyses were performed with SPSS software (SPSS, Chicago, IL). P values < 0.05 were considered to be significant.

## Supplementary information

Supplementary Figures

## Data Availability

All data analyzed during this study are included in this published article and its Supplementary Information files.
