# Supplementary material for: Connective tissue growth factor is correlated with peritoneal lymphangiogenesis
Source: Sci Rep. 2019 Aug 21;9:12175. doi: 10.1038/s41598-019-48699-9 (PMC6704065; doi:10.1038/s41598-019-48699-9)
Supplement: Supplementary file 1 — Supplementary Figures [file 41598_2019_48699_MOESM1_ESM.pdf]

Connective tissue growth factor is correlated with peritoneal lymphangiogenesis

Hiroshi Kinashi[1,2], Naohiro Toda[3], Ting Sun[4], Tri Q. Nguyen[2], Yasuhiro Suzuki[4], Takayuki Katsuno[1], Hideki Yokoi[3], Jan Aten[5], Masashi Mizuno[4], Shoichi Maruyama[4], Motoko Yanagita[3], Roel Goldschmeding[2], and Yasuhiko Ito[1]

[1] Department of Nephrology and Rheumatology, Aichi Medical University, Nagakute, Japan
[2] Department of Pathology, University Medical Center Utrecht, Utrecht, The Netherlands
[3] Department of Nephrology, Graduate School of Medicine, Kyoto University, Kyoto, Japan
[4] Department of Nephrology and Renal Replacement Therapy, Nagoya University Graduate School of Medicine, Nagoya, Japan
[5] Department of Pathology, Academic Medical Center, University of Amsterdam, Amsterdam, The Netherlands

Supplementary Figures 1-7

Pre-PD uremia

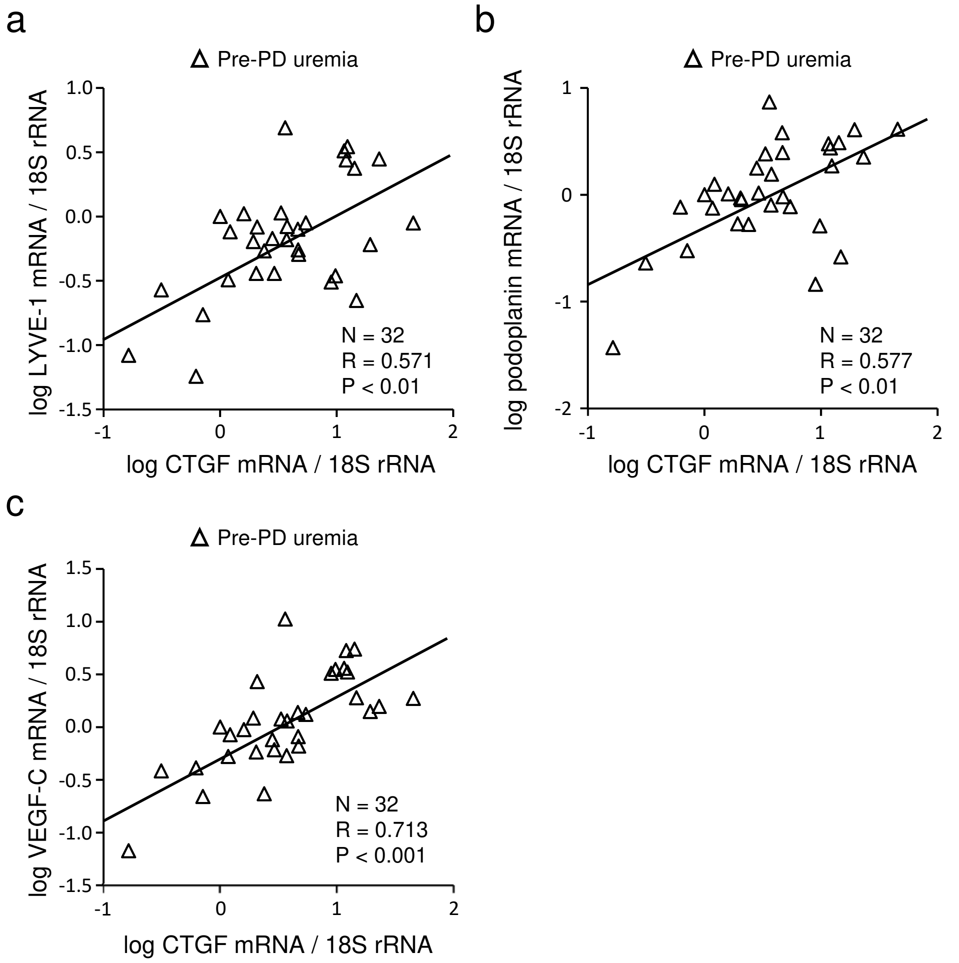

Supplementary Figure 1. Connective tissue growth factor (CTGF) expression was correlated with expression of lymphatic markers and vascular endothelial growth factor-C (VEGF-C) in human peritoneal biopsies derived from uremic patients before initiation of peritoneal dialysis (PD).
Quantitative polymerase chain reaction analysis showed positive correlations between CTGF messenger RNA (mRNA) expression and lymphatic vessel endothelial hyaluronan receptor-1 (LYVE-1) (a), podoplanin (b), and VEGF-C (c) mRNA expression in human peritoneal biopsies before initiation of PD. 18S ribosomal RNA (rRNA) was used as an internal reference. Values were transformed into the logarithmic scale for Pearson correlation.

PD without UFF

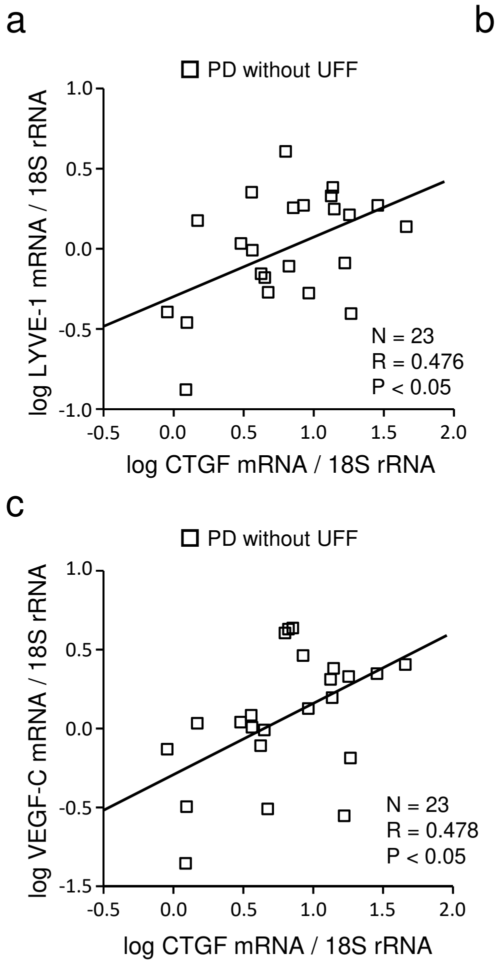

Supplementary Figure 2. Connective tissue growth factor (CTGF) expression was correlated with expression of lymphatic markers and vascular endothelial growth factor-C (VEGF-C) in human peritoneal biopsies from patients undergoing peritoneal dialysis (PD) not complicated by ultrafiltration failure (UFF).

Quantitative polymerase chain reaction analysis showed positive correlations between CTGF messenger RNA (mRNA) expression and lymphatic vessel endothelial hyaluronan receptor-1 (LYVE-1) (a), podoplanin (b), and VEGF-C (c) mRNA expression in human peritoneal biopsies from PD patients without UFF. 18S ribosomal RNA (rRNA) was used as an internal reference. Values were transformed into the logarithmic scale for Pearson correlation.

PD with UFF

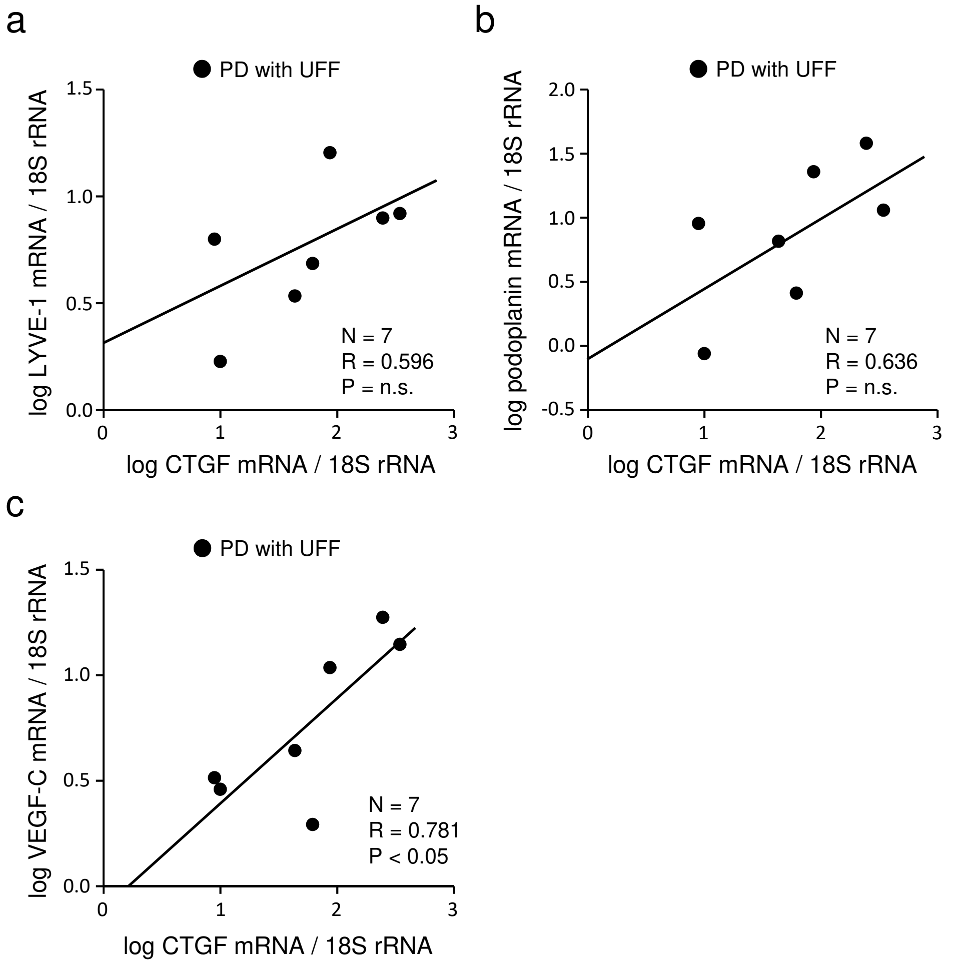

Supplementary Figure 3. Connective tissue growth factor (CTGF) expression was correlated with vascular endothelial growth factor-C (VEGF-C) expression in human peritoneal biopsies from patients undergoing peritoneal dialysis (PD) complicated by ultrafiltration failure (UFF).

CTGF messenger RNA (mRNA) expression tended to be correlated with lymphatic vessel endothelial hyaluronan receptor-1 (LYVE-1) (a) and podoplanin (b) mRNA expression in human peritoneal biopsies from PD patients with UFF, but the relationship was not statistically significant. (c) CTGF mRNA expression was significantly correlated with VEGF-C mRNA expression in the UFF peritoneum. 18S ribosomal RNA (rRNA) was used as an internal reference. Values were transformed into the logarithmic scale for Pearson correlation. n.s.; not significant.

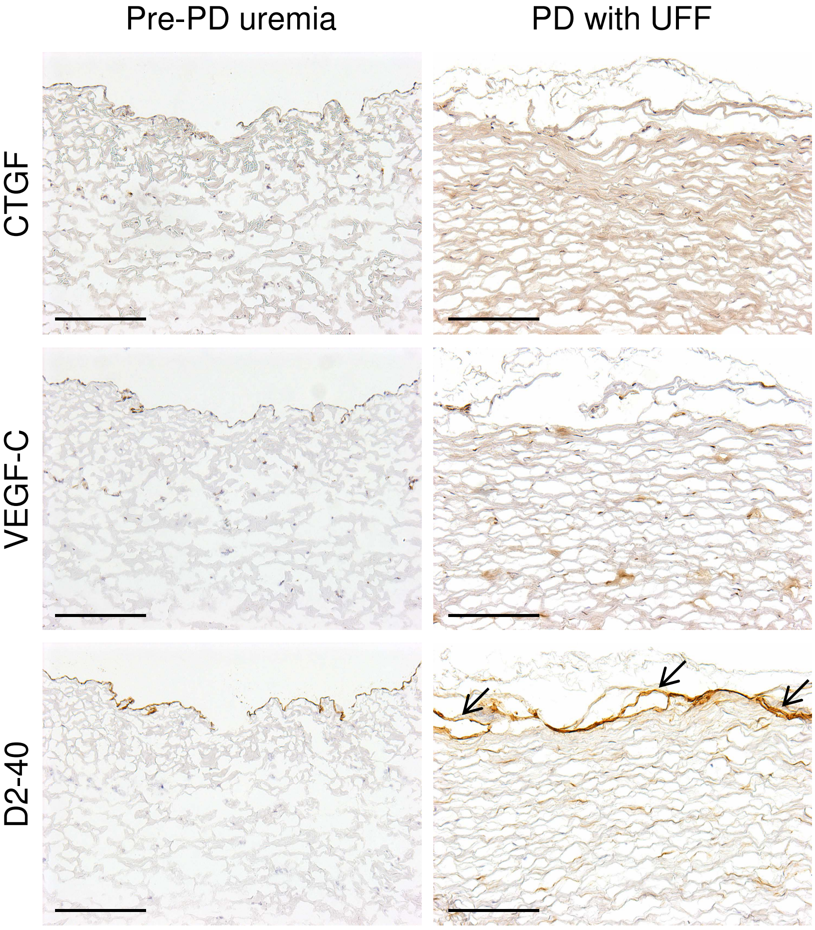

Supplementary Figure 4. Connective tissue growth factor (CTGF), vascular endothelial growth factor-C (VEGF-C), and D2-40 expression were increased in the peritoneum from a patient undergoing peritoneal dialysis (PD) complicated by ultrafiltration failure (UFF) compared with the peritoneum from a uremic patient before initiation of PD.
Human peritoneal biopsy specimens were collected from a pre-PD uremic patient at the time of PD catheter insertion and from a PD patient with UFF at the time of PD catheter removal. Immunohistochemistry (IHC) for CTGF, VEGF-C, and D2-40 was performed on consecutive sections. Representative IHC images showed that the increased expression of CTGF was associated with the increased expression of VEGF-C and D2-40-positive lymphatic vessels in the UFF peritoneum. Arrows indicate D2-40-positive lymphatic vessels. Scale bars; 200 μm.

IHC grading in rat diaphragm

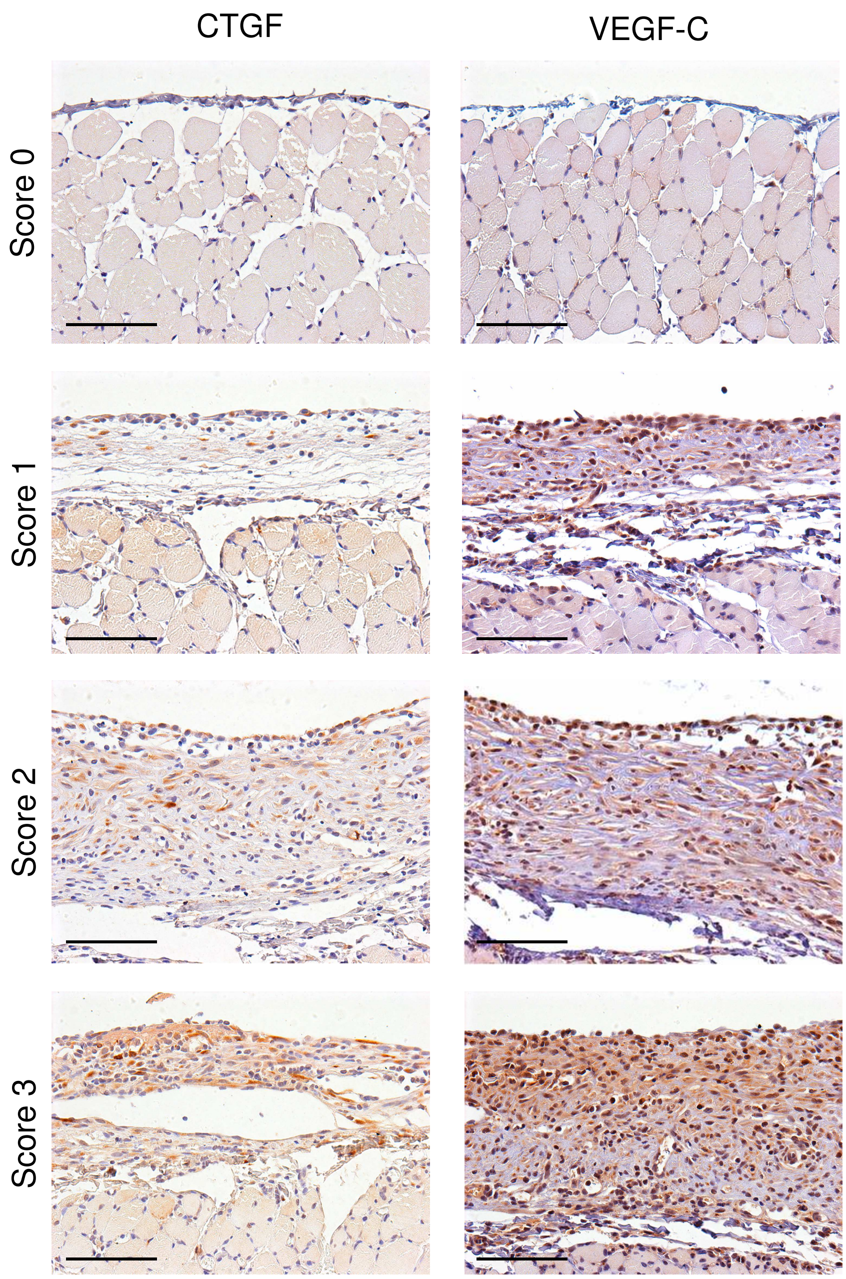

Supplementary Figure 5. Representative images of immunohistochemical (IHC) grading for connective tissue growth factor (CTGF) and vascular endothelial growth factor-C (VEGF-C) in a rat model of diaphragmatic fibrosis.

Diaphragmatic fibrosis was induced by intraperitoneal injection of chlorhexidine gluconate (CG) in rats. Control rats were treated with saline. Representative images showed the staining intensity for IHC scores as follows: 0, absent; 1, mild; 2, moderate; 3, extensive. Scale bars; 100 μm.

IHC grading in mouse peritoneum

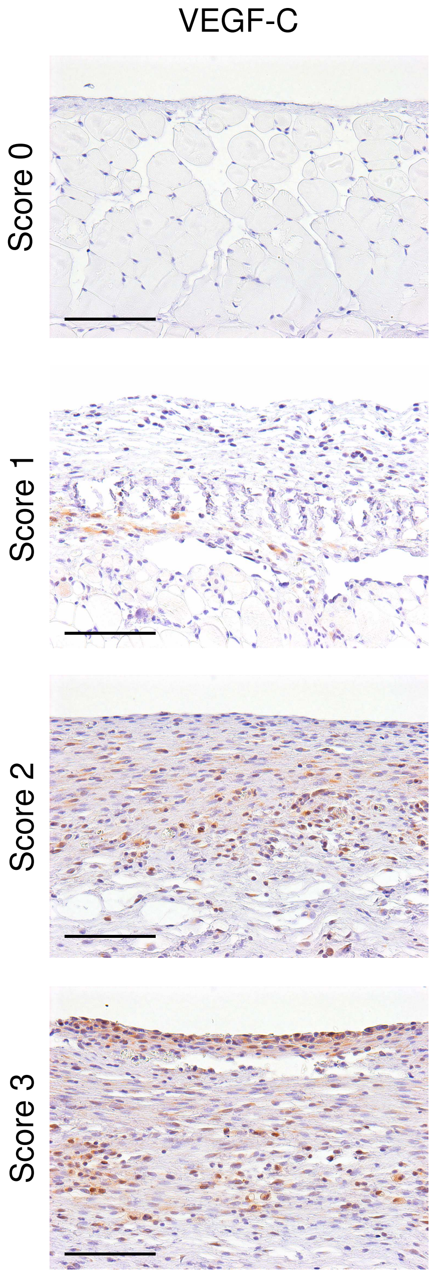

Supplementary Figure 6. Representative images of immunohistochemical (IHC) grading for vascular endothelial growth factor-C (VEGF-C) in a mouse model of peritoneal fibrosis.
Peritoneal fibrosis was induced by intraperitoneal injection of chlorhexidine gluconate (CG) in mice. PBS-treated mice were used for comparison. Representative images showed the staining intensity for VEGF-C scores as follows: 0, absent; 1, mild; 2, moderate; 3, extensive. Scale bars; 100 μm.

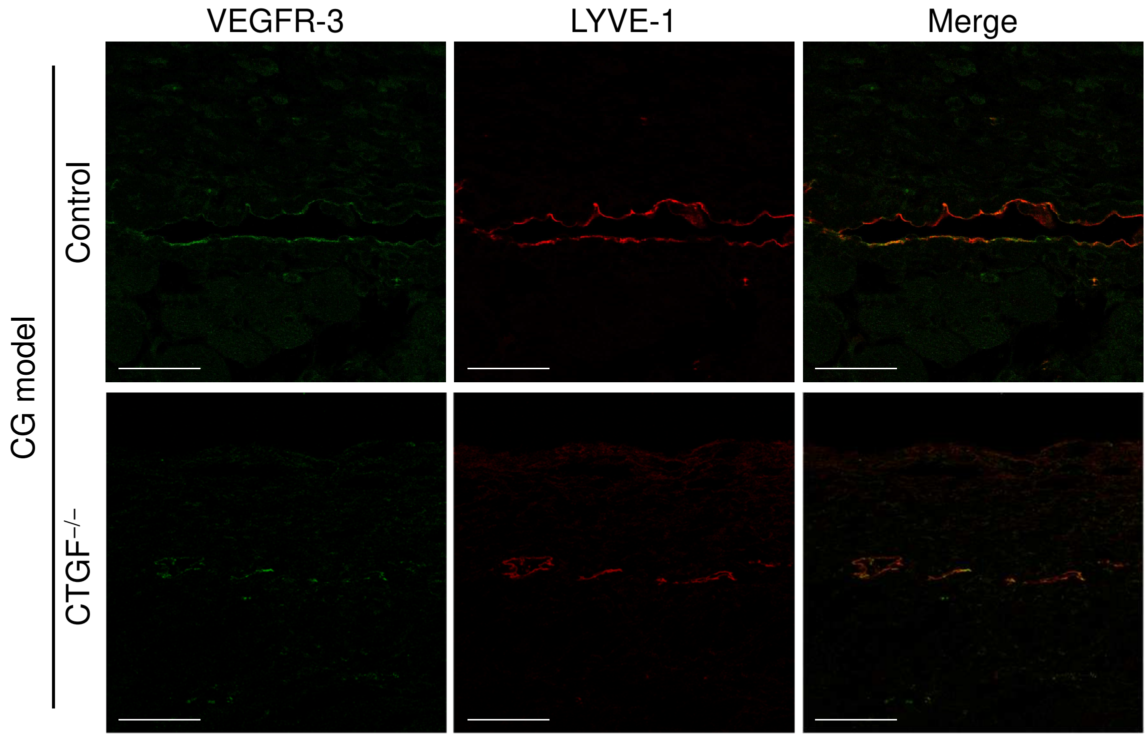

Supplementary Figure 7. Double immunofluorescent staining for vascular endothelial growth factor receptor-3 (VEGFR-3) and lymphatic vessel endothelial hyaluronan receptor-1 (LYVE-1) in a mouse model of peritoneal fibrosis.

Peritoneal fibrosis was induced by intraperitoneal injection of chlorhexidine gluconate (CG) in wild-type mice (Control) and CTGF knockout (CTGF$^{-/-}$) mice. Double immunofluorescent staining for two lymphatic markers showed the similar expression pattern between VEGFR-3 and LYVE-1 in the peritoneal fibrosis of both control mice and CTGF$^{-/-}$ mice. Scale bars; 50 μm.
